# A new Ingolfiellid (Crustacea, Amphipoda, Ingolfiellidae) from an anchialine pool on Abd al Kuri Island, Socotra Archipelago, Yemen

**DOI:** 10.3897/zookeys.302.5261

**Published:** 2013-05-20

**Authors:** Valentina Iannilli, Ronald Vonk

**Affiliations:** 1 ENEA, Technical Unit of Sustainable Management of Agro-Ecosystems, C.R. Casaccia via Anguillarese 301, 00123 Rome, Italy; 2 Museo Civico di Storia Naturale di Verona, Lungadige Porta Vittoria, 9, I-37129 – Verona, Italy; 3 Naturalis Biodiversity Center, Darwinweg 2, P.O. Box 9517, 2300 RA Leiden, The Netherlands; 4 Institute for Biodiversity and Ecosystem Dynamics, University of Amsterdam, Amsterdam 1098 XH, The Netherlands

**Keywords:** Taxonomy, meiofauna, subterranean thalassoid amphipods, beach environment, mesopsammon, Arabian Sea

## Abstract

*Ingolfiella arganoi* **sp. n.** from Abd al Kuri Island in the Arabian Sea is described from two specimens, a male and a female. The western shore of the Indian Ocean was hitherto a vacant spot in the distribution of circumtropical shallow marine interstitial ingolfiellids and therefore the location of the new species fills a meaningful gap in the geography of the family. Morphologically, the new species shows close affinities with *Ingolfiella xarifae* from the Maldives.

## Introduction

**Figure 1.**
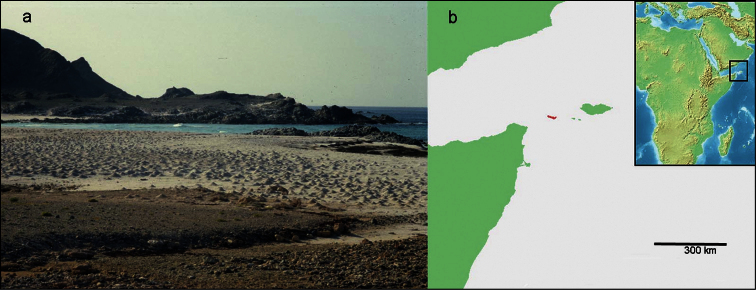
**a** Abd al Kuri, beach, with gully in foreground, scoured in the sand by rainwater during the wet season (photograph taken while standing at the collection site by R. Argano). Pointed hills on the white beach sand are *Ocypode* crab burrows **b** position of Abd al Kuri Island (red color) in the Arabian Sea.

During sampling of aquatic fauna from the Socotra Archipelago conducted by Roberto Argano and co-workers (Taiti and Ferrara 2004), two specimens of a new species of *Ingolfiella* were found in the mesopsammon of an anchialine pool on Abd al Kuri Island (Fig. 1a, b). Anchialine water bodies are well known to offer a great potential of unique species (Becking et al. 2011 and references herein). Ingolfiellid amphipods are sporadically found and are confined to fresh, brackish, and marine ground- and cave waters, and even ocean floor habitats (Ruffo 1950; Spooner 1959, 1960; Vonk and Schram 2003). They are seldom observed in great numbers and the specimens from the small island of Abd al Kuri are no exception to this rule; only two specimens were found. Their discovery points to a long-awaited geographic link in the chain of locality records in this group, as they bridge a gap in the so often presumed Tethys distribution. Until now, no ingolfiellid has been reported between the coasts of Greece in the west and the beaches of the Maldives in the east – a stretch well over 6000 km.

The late Sandro Ruffo, curator of the natural history museum of Verona, started the work on the two specimens from Abd al Kuri, helped by one of us (V.I.), but did not finish it because he was not confident about the status of a new species based on so little material and the presence of only few available distinctive morphological characters. A few years later we decided to take up this work and bring it to fruition because it provides relevant new information on the geographic range of ingolfiellids and on new combinations of character states as shown by these specimens from an anchialine environment in the Arabian Sea.

## Material and methods

The two specimens were collected on a beach on the north coast of Abd al Kuri Island, Republic of Yemen, (12°11.988'N, 52°15.943'E), close to a little village called Bait Eesa. Sampling was done with a micro-creel in coarse sand at the bottom of a pool in an otherwise dry wadi, running as a slight depression to the sea (pers. comm. R. Argano). The approximate position of the pool with unknown salinity from which the ingolfiellids were collected on 7 February 2000 is shown in Fig. 1a.

## Taxonomy

### Order AMPHIPODA Latreille, 1802 Suborder INGOLFIELLIDEA Hansen, 1903 Family Ingofiellidae Hansen, 1903 Genus *Ingolfiella* Hansen, 1903

#### Ingolfiella arganoi sp. n.

urn:lsid:zoobank.org:act:807BB2C1-62F5-4930-A55B-74AF21C033EB

http://species-id.net/wiki/Ingolfiella_arganoi

Figs 2 –5

##### Material examined.

Two specimens: one male **holotype**, 1.4 mm, dissected and mounted in Faure’s liquid on slide MSNVRCr nr. 434; one preparatory female **paratype** on two slides MSNVRCr nr. 470 in Museo di Storia Naturale di Verona, Italy.

##### Diagnosis.

Lateral lobes on frontal margin of head developed. Maxillule, basal endite (= outer lobe) left and right with asymmetrical seta. Gnathopods 1 and 2 carpochelate with oblique palm, dactyli with a serrated inner margin with four teeth. Female with extra palmar margin robust seta. Oöstegites on pereiopod 3 and 4, with three regularly placed small button-like processes. Gills present on P3-5. Dactylus of P3 and P4 with slender trifid unguis; P5-7 with thicker bifid unguis, not clearly separated from dactylus. Pleopods 1-3 subtrapezoidal and similar, except first pleopod in male which is flexed and has a broadened tip. Uropod 1 with inner ramus about 1.5 times as long as outer ramus; uropod 2 peduncle without basofacial spine and with two diagonal rows of sturdy rectangular setae, three rows in female, individual setae mostly bifid but with some of them trifid at the tip.

##### Etymology.

The new species is named after Roberto Argano (University of Rome “la Sapienza”) who collected the specimens and gave them to the Verona Museum for study.

##### Description.

**Figure 2.**
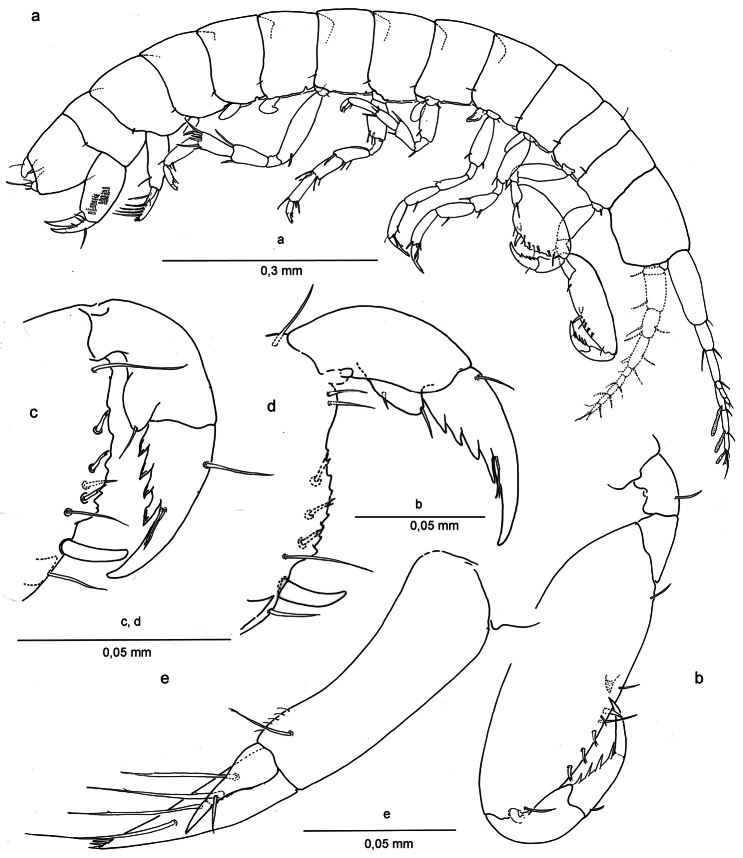
*Ingolfiella arganoi* sp. n., male holotype 1.4 mm. **a** habitus, male 1.4 mm **b** left gnathopod I, lateral **c** right gnathopod II, lateral **d** left gnathopod II, medial **e** right uropod I, lateral.

*Body* elongate, without coloration, all segments laterally compressed. *Head* (Fig. 2a) with lateral margin rounded; lateral or ‘ocular lobes’ present on frontal margin, well developed, suboval. *Pleonites* I-III with diffusely developed posteriorly rounded epimeral plates adorned with simple seta, a superficial marginal edge slightly visible. *Urosomite* III subcilindrical, slightly longer than deep, enclosing base of telson and uropod III.

*Antennule* (Fig. 2a), peduncular article slightly shorter than head; article ratio 1:0,42:0,42; flagellum of 4 articles, half the length, articles 2-4 with 1 aesthetasc; accessory flagellum slightly shorter than flagellar articles 1+2, three articles.

*Antenna* (Fig. 2a) subequal in length to antennule; flagellum of 5 articles, slightly shorter than half the length of peduncle, the last article bearing one aesthetasc (antenna drawn by S. Ruffo but not present in mounted slides).

*Mandibles* with non-triturative molar process, spiniform. Left mandible (Fig. 5d) with broad incisor, right mandible (Fig. 5c) with fine serrations on lacinia and molar process margin.

*Maxillule* (Figs 5a, e) coxal endite (= inner lobe) with 3 simple setae; basal endite (= outer lobe) with six robust setae of which the second one on the medial side has four teeth in the left maxillule and three teeth in the right one. Endopod (= palp) two-segmented, distal segment with two setae.

*Maxilla* (Fig. 5b) with short, equally long plates, each bearing four distal setae.

*Maxilliped* (Fig. 5f) basal endite slender, with one simple seta; ischium with two setae; merus and carpus without setae; propodus with one seta; dactylus with one lateral robust seta and distally two long setae, unguis not discernible.

**Figure 3.**
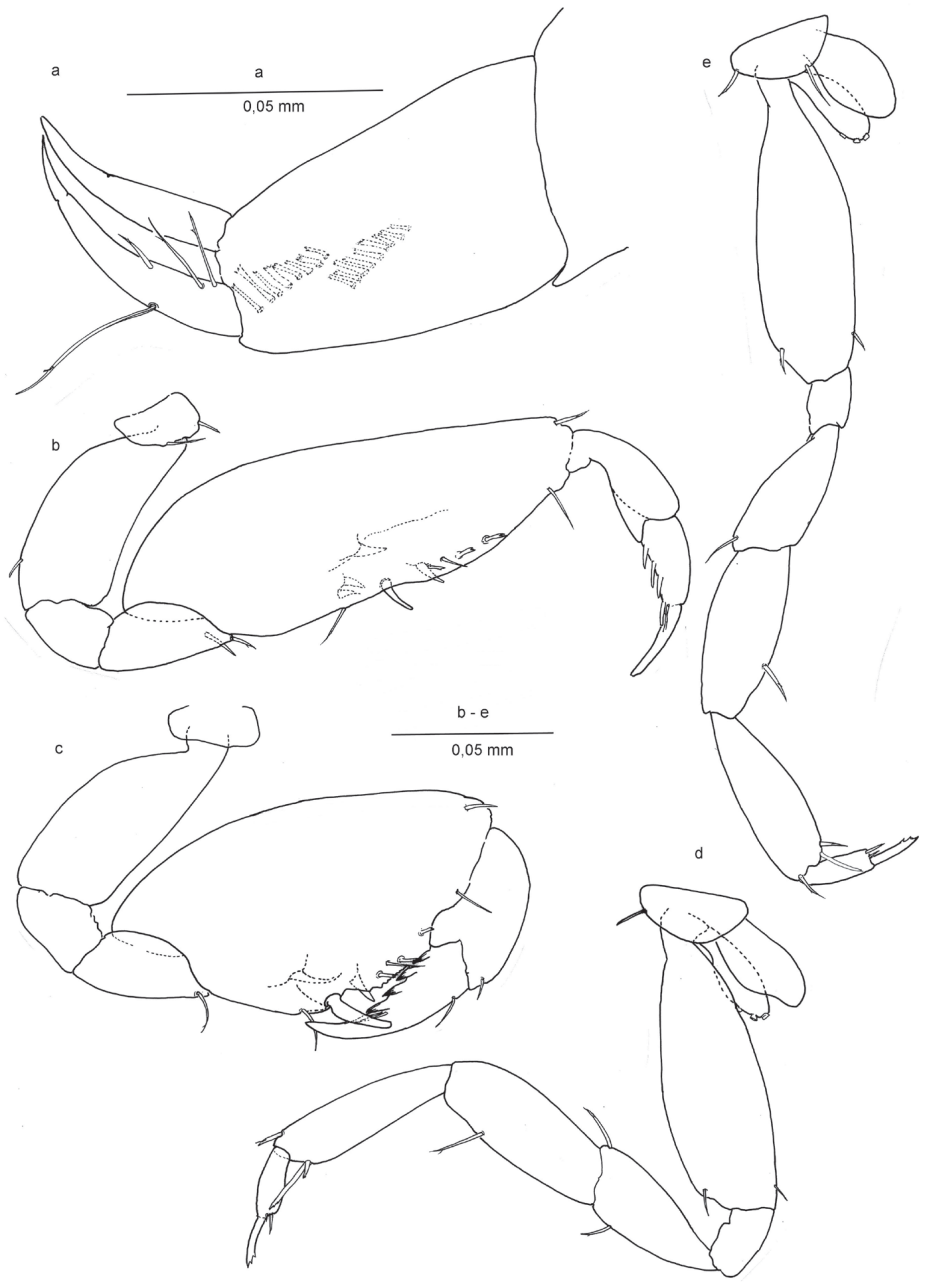
*Ingolfiella arganoi* sp. n., male holotype 1.4 mm. **a** right uropod II, lateral; female paratype 1.5 mm. **b** right gnathopod I, lateral **c** right gnathopod II, lateral **d** right pereiopod III, lateral **e** right pereiopod IV, lateral.

*Oöstegites* on pereiopods III-IV (Figs 3d, e), suboval, without setae and with 3 button-like processes.

Coxal gills on pereiopods III-V.

*Gnathopod I* (Figs 2b, 3b) carpo-subchelate, palm strongly oblique, carpus 2.4 times as long as wide, palm margin smooth, not serrated, and with three short, bifid flagellate setae along lateral side of margin, and one simple seta on palm angle in male. In female two of such setae of which one placed closer to the row of three bifid setae. Just posterior to the palmar angle seta is a broad triangular spine on the medial side in the male, and three spines in the female: two smaller ones and a larger, more pointed one. Dactylus with four long spines along posterior margin and thin setules or grooves at the base of the unguis.

*Gnathopod II* (Figs 2c, d; 3c) Carpo-subchelate, palm oblique, carpus stronger than in gnathopod I, subtrapezoidal, carpal index = 4.6, palm angle defined by one large seta and one smaller spine in female (Fig. 3c), one seta in male (Figs 2c, d), and with triangular tooth proximal to the palmar angle seta, palm margin with irregular serrations; propodus strong with lobe on lateral side ending in a setule, less pronounced in female; dactylus with four strong teeth enforced with thick margins on lateral side and a groove or bundled setules at the base of the unguis.

*Pereipods III*-*IV* (Figs 3d, e) with two distal setae on dactylus at the base of the unguis, and three distal setae on propodus, one of them long and apically bifid, unguis apically trifid. Oöstegites with in both pereiopods regularly placed series of 3 button-like processes.

**Figure 4.**
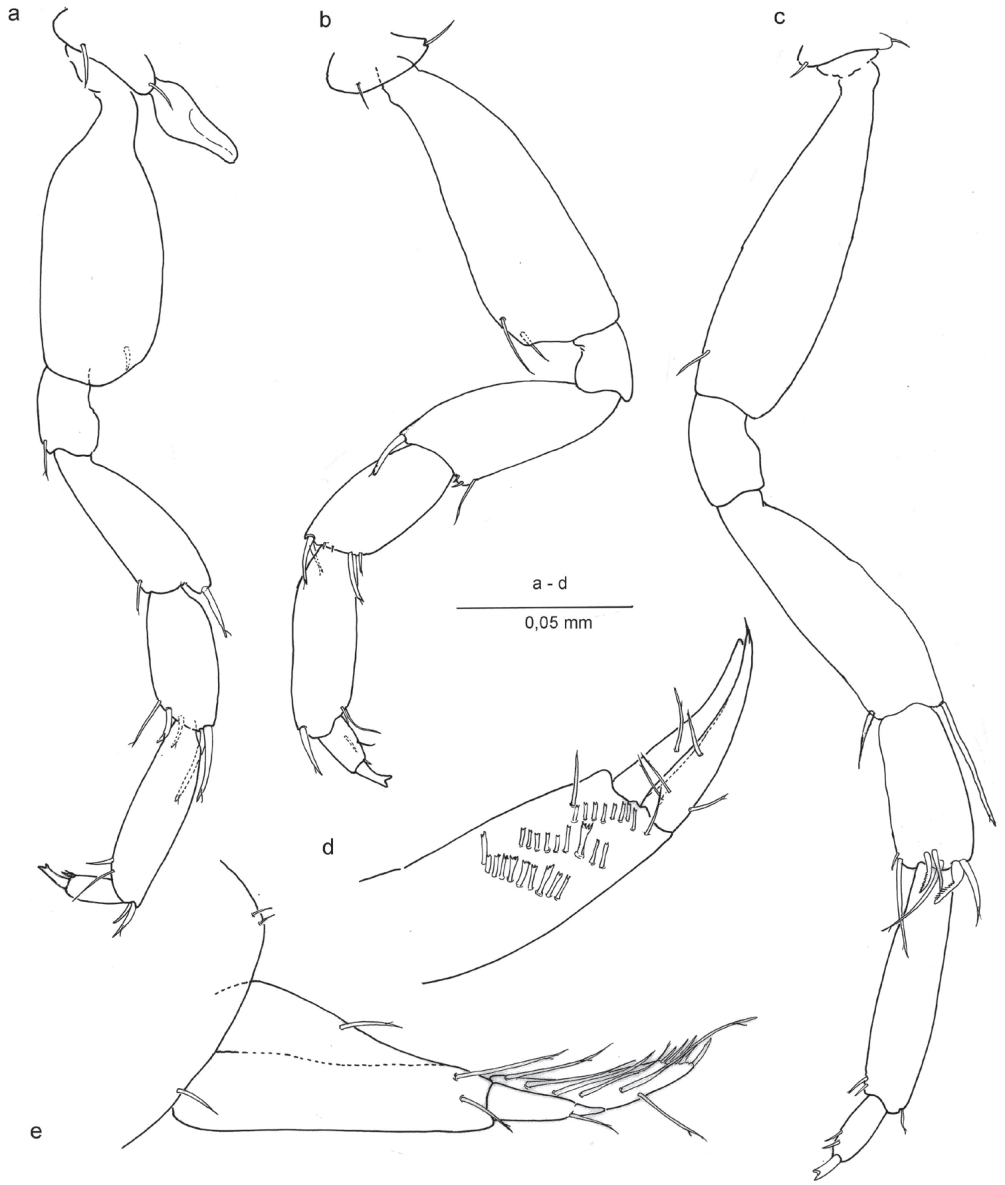
*Ingolfiella arganoi* sp. n., female paratype. **a** pereiopod V **b** pereiopod VI **c** pereiopod VII **d** right uropod II, medial **e** left uropod I, lateral.

*Pereiopods V – VII* (Figs 4a,b, c) progressively longer towards P7; basis of P5 broad, that of P7 slender; carpus of P5 with two long and stout distal setae, others shorter; carpus of P7 with broad, curved and modified comb setae; merus of P7 with long distal seta; dactyli with two small setae distally; unguis bifid.

*Pleopods I-III* (Fig. 2a) subtrapezoidal, without setae. Pleopod I in male deformed or broadened distally.

*Uropod* I (Figs 2e, 4e) male: protopod with one seta and a row of fine setules on anterolateral margin; exopod with very feeble segment suture and one seta placed at two-thirds the length; endopod with terminal row of spines and four long setae laterally. In female protopod with three setae; endopod with six long setae laterally.

*Uropod* II (Figs 3a, 4d), protopod with two oblique comb rows in male, and three in female; setae of rows more or less rectangular with bifid or trifid, or even comb-like tips; endopod slightly longer than exopod, sharper, and with four setae.

*Uropod* III (Fig. 2a) short, 2 segmented, with one ramus, protopod with 2 distal setae, ramus short with 1 distal seta.

Telson (Fig. 2a) globose, with 1 pair of long dorsal setae.

Differences between male and female: gnathopods without extra palmar seta in male, and uropod II without a third comb row in male. Pleopod I in the male has a broadened tip.

**Figure 5.**
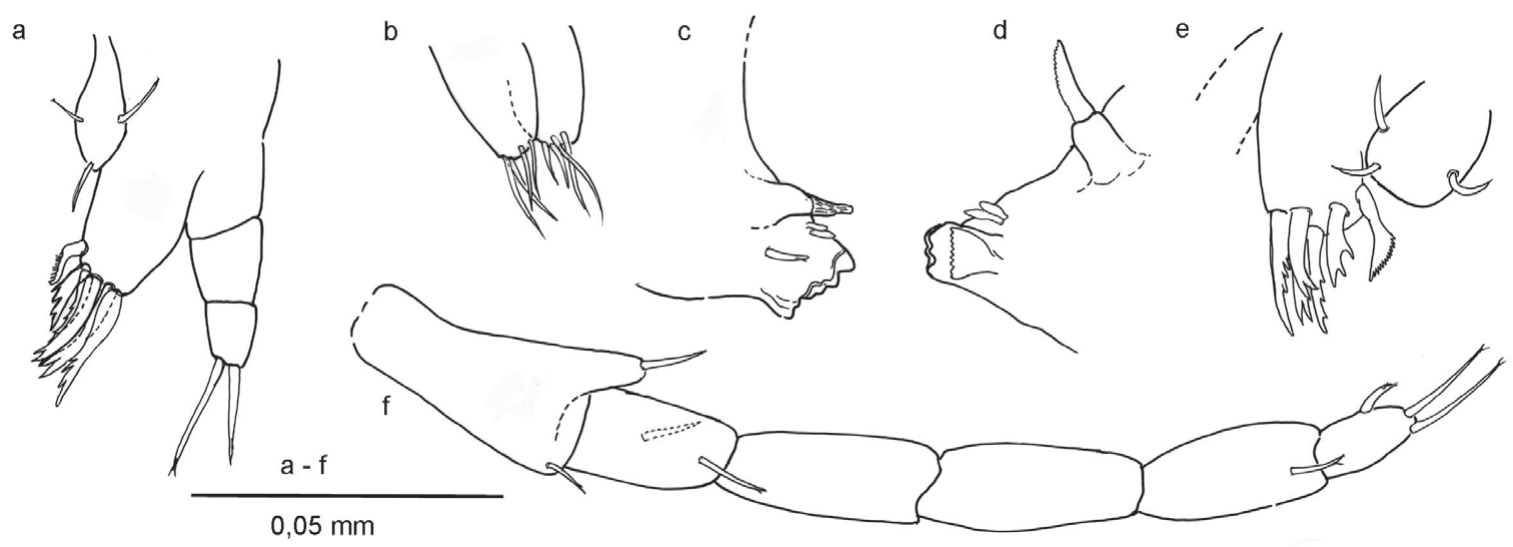
*Ingolfiella arganoi* sp. n., female paratype. **a** left maxillule **b** maxilla **c** right mandible **d** left mandible **e** right maxillule **f** right maxilliped.

##### Remarks.

*Ingolfiella arganoi* sp. n. shares most morphological character states with a species found 2500 km southeastward across the Indian Ocean, on the Maldives, namely *Ingolfiella xarifae* (Ruffo, 1966), from washed-out broken coral pieces (*Favites* sp.). Species ranges of stygobionts have not been reported to exceed such large distances in the past and molecular work on cryptic lineage diversity of populations of groundwater crustaceans have even diminished existing ranges to distances of less than 1000 km (Trontelj et al. 2009).

For now, five clear morphological differences justify the designation of a new species that also is geographically quite far away from its nearest congeners. These differences can be observed in the four spines on the medial margin of the propodus of gnathopod I – three spines in *Ingolfiella xarifae*; subtrapezoidal carpus of gnathopod 2 – elongate oval in *xarifae*; palmar index of gnathopod 2 is 4.6 – against 6.4 in xarifae; palm of gnathopod 2 strongly serrate – almost smooth in *xarifae*; pereiopod VII with specialized, combed, robust setae distally on carpus – not present in *xarifae*.

The differences in the placement, form and number of setae have shown to be quite consistent in the case of the poorly setose ingolfiellids (Vonk and Sanchez 1991). The spines and setae that have remained, are perhaps critically functional due to selective reductive factors in the underground environment.

The oöstegites have 3 small button-like processes. The same character was described in *Ingolfiella alba* Iannilli et al., 2008, where also three small button-like processes are present, but here from P3 to P5. Re-examination of *Ingolfiella xarifae* typus by Sandro Ruffo and V.I. allowed them to observe also on the oöstegites of this species the button-like processes, as described for *Ingolfiella alba*.Something similar was described in *Metaingolfiella mirabilis* Ruffo, 1969, although the processes were smaller and more numerous. These structures are probably present in other *Ingolfiella* species but have yet to be observed and described (Iannilli et al., 2008). Stock (1979) observed in *Ingolfiella quadridentata* Stock, 1979, that oöstegites found on P3 and P4 were: “curved, truncate at tip, provided with 3 apical teeth, but without setae.”

In other amphipods with preparatory females (females in a moult stage in between two brooding periods), these structures are sometimes present on the oöstegites (pers. comm. D. Jaume). Slattery (1985) mentions the development of oöstegites in infaunal amphipod families of Phoxocephalidae and Haustoriidae undergoing three stages of development: buds (of the oostegite itself), preparatory (moderately long oöstegites with some setae), and mature (long oöstegites, curved, and setose to form a brood-carrying pouch or marsupium). If the buttons, that occur in threesomes on the oöstegites of pereiopods III and IV in *Ingolfiella arganoi*, can be observed in other ingolfiellids as being present at the same time with setae this might prove their precursory role. On the other hand, in contradiction to these observations, are our studies of abundant material of *Ingolfiella alba*, that shows this character. The material consists of several individuals collected in different years (from 1992 to 2004) and in different months of the year. We could verify that the structure is always the same, namely the presence of only three button-like processes, and we did not find setae on the oöstegites. So an interpretation of these structures being preparatory setae seems not convincing for *Ingolfiella* species.

In agreement with Ruffo and Vigna Taglianti 1989 the new species could be placed in the subgenus *Tethydiella*, although Vonk and Schram (2003), basing their phylogeny on more characters, did not use the splitting of *Ingolfiella* into genus and subgenera as several taxa are poorly described when compared to recent taxonomic descriptions and only few species have both sexes well known.

However, the species-groups in sensu Ruffo (1970) and Ruffo and Vigna Taglianti (1989) may still be of practical taxonomic use and the geographical location as well as the morphological diagnosis fits the *Tethydiella* group. Of course, more detailed biogeographic data are required to reconstruct the history of the actual distribution of Ingolfiellidae (Iannilli et al. 2008).

## Subterranean thalassoid crustaceans of the region

The present discovery represents the westernmost record of ingolfiellids in the Indo-Pacific Oceans. The Red Sea shores, Gulf of Aden, and the shores of the Arabian sea are relatively unknown areas for stygobiont and marine interstitial crustaceans. Geographically it is an interesting region as it forms the corridor between the better known Mediterranean and south east Asian/Australian stygofaunas. Sampling by Stock and Nijssen (1965) on Entedebir Island in the southern Red Sea revealed the presence of the circumtropical marine groundwater amphipod genus *Psammogammarus* (Vonk and Stock 1987; Vonk 1990; Van der Ham and Vonk 2003; Tomikawa et al. 2010; Vonk et al. 2011), suggesting the possible occurrence of the usual inhabitants of the present coastal groundwater biotope. In this respect also *Angeliera xarifae* Siewing, 1959 can be mentioned. This is a microparasellid isopod, sampled from coastal groundwater of Abd al Kuri during the Xarifa expedition of 1957 (Wallace and Zahir 2007). In 2005 Iannilli et al. described the bogidiellid amphipod *Nubigidiella theresiae*, from a freshwater well at Bin Aissa, on Abd al Kuri Island. Until 2005 the only amphipod species known from the subterranean waters of Socotra was *Indoweckelia superstes* Holsinger & Ruffo, 2002, from a water well on the main island of Socotra. These are mainly freshwater subterranean amphipods with well investigated marine affinities. Recently the coastal aquifer waters of Oman showed to contain *Metacrangonyx* (Jaume & Vonk, 2012), a stygobiont amphipod with proven marine origins (Bauzà-Ribot et al. 2012), distributed in marine and freshwaters from the Caribbean to its current eastern border, the Indian Ocean. On Socotra and Abd al Kuri new cyclopoid copepods were described from brackish wells and caves (Mirabdullaev et al. 2002) showing the island’s isolated status. The first inland, freshwater ingolfiellid of the region, as well as those from deep marine sediments, are still to be discovered.

## Supplementary Material

XML Treatment for Ingolfiella arganoi
